# Serum Levels of Coagulation Factors in Patients with Inflammatory Bowel Disease

**DOI:** 10.30699/IJP.2025.2061251.3460

**Published:** 2025-11-11

**Authors:** Bahram Memar, Ali Moradi, Shohreh Khatami, Hassan Vosoughinia, Mitra Ahadi

**Affiliations:** 1 Department of Pathology, School of Medicine, Mashhad University of Medical Sciences, Mashhad, Iran; 2 Clinical Research Development Unit, Ghaem Hospital, Mashhad University of Medical Sciences, Mashhad, Iran; 3 Orthopedic Research Center, Mashhad University of Medical Sciences, Mashhad, Iran; 4 Department of Internal Medicine, Faculty of Medicine, Mashhad University of Medical Sciences, Mashhad, Iran; 5 Department of Gastroenterology and Hepatology, School of Medicine, Mashhad University of Medical Sciences, Mashhad, Iran

**Keywords:** Inflammatory bowel disease, IBD, Crohn disease, Ulcerative colitis, Blood Coagulation Factors.

## Abstract

**Background & Objective:**

Inflammatory bowel diseases (IBD) is described by increased coagulability and prothrombotic state and can be associated with coagulopathies. Although many causes of increased coagulability and thrombosis have been reported in IBD, there is no definitive evidence for most of them. This study aimed to define the changes in Blood Coagulation Factors in patients with IBD compared to healthy controls.

**Methods:**

In this case-control study, serum levels of protein C, protein S, antithrombin III, fibrinogen, and Homocysteine were evaluated in 59 patients with a confirmed IBD, (23 with Crohn disease and 36 with ulcerative colitis) (case group) and 29 healthy individuals (control group) matched for age and gender.

**Results & Conclusion:**

Significant differences were found in all five studied markers between IBD and non-IBD patients (protein C (P=0.033), protein S (P=0.006), antithrombin III (P<0.001), fibrinogen (P=0.016) and Homocysteine ​​(P<0.001)), however, multivariate analysis showed a significant role for only Homocysteine (OR=0.957, 95%CI: 0.93-0.986, P=0.003) in predicting IBD. Regarding the results, it can be alleged that despite the significant difference in the level of Blood Coagulation Factors between the IBD and non-IBD patients, only the serum level of Homocysteine has a predictive role for IBD.

## Introduction

In recent years, the prevalence of inflammatory bowel diseases (IBD), particularly Crohn disease, has increased significantly in regions historically considered low-risk, including Iran. This rise points to environmental factors as important contributors to disease development. Iranian studies from 1969 to 2002 have documented IBD cases, which primarily include ulcerative colitis and Crohn disease—both chronic inflammatory conditions of the gastrointestinal tract diagnosed through clinical, pathological, endoscopic, and radiological assessments (1). The modernization of lifestyle and dietary habits in Iran, consistent with the cold chain theory, may explain the delayed emergence of IBD in comparison to Western nations (2).

IBD is linked to a hypercoagulable state, placing patients at a markedly increased risk for venous thromboembolism (VTE). Systematic reviews by Yuhara et al. and Nguyen et al. revealed that individuals with IBD face a two- to threefold higher VTE risk than healthy controls. Deep vein thrombosis (DVT) and pulmonary embolism are most common, though emboli in cerebral, portal, hepatic, retinal, and mesenteric veins also occur, albeit less frequently (3-5). The VTE in IBD is characterized by high recurrence rates and correlations with disease activity. Contributing mechanisms include inflammation, endothelial dysfunction, platelet abnormalities, coagulation system activation, and impaired fibrinolysis (6).

While the etiology of IBD remains unclear, environmental exposures, genetic predisposition, and immune dysregulation are widely accepted contributors (7, 8). Chronic intestinal inflammation also increases oxidative stress. Rana et al. demonstrated elevated oxidative markers and diminished glutathione in ulcerative colitis patients, suggesting a potential role in disease recurrence despite treatment (9). 

Recent progress in understanding genetic influences on thrombosis has extended to IBD. Although genetic risk factors for clot formation are not common in IBD, their presence significantly elevates the risk of VTE. For example, Factor V Leiden (FVL), a major VTE risk factor, has shown similar prevalence in IBD patients and healthy populations (10). Research on methylenetetrahydrofolate reductase (MTHFR) polymorphisms has produced inconsistent results. A systematic review found no direct link between MTHFR variants and IBD, although folate deficiency in affected individuals may increase thrombosis risk (11). 

Despite numerous studies on thrombosis in IBD, the underlying drivers of elevated VTE incidence remain inconclusive (6). Some data suggest that reduced serum levels of protein C, protein S, and antithrombin III, and elevated levels of homocysteine and fibrinogen, may signal increased coagulability (12, 13). 

This study aims to explore the serum levels of blood coagulation factors, namely protein C, protein S, antithrombin III, fibrinogen, and homocysteine, in IBD patients and compare them to healthy controls. Focused on Iranian patients and their genetic background, the findings may enhance understanding of VTE pathogenesis and support improved prevention strategies, particularly for hospitalized individuals at high thrombotic risk.

## Materials and Methods

This retrospective cross-sectional study evaluated This observational case-control study was conducted at the IBD specialty clinic of Ghaem Hospital between 2017 and 2018. A total of 59 patients with confirmed inflammatory bowel disease (Crohn disease or ulcerative colitis) and 29 healthy controls were enrolled. Based on serum protein C levels reported by Dolapcioglu et al. (14), sample size was calculated using α = 0.05, 80% power, and a 2:1 case-to-control ratio, with adjustments made to accommodate dropouts. 

### Procedure

All participants were informed about the study objectives and provided with written consent. Demographic data—age, gender, and IBD classification—were collected using structured checklists. Under standardized conditions, 10 ml of blood was drawn from each subject by a single operator to ensure procedural consistency. Samples were centrifuged (2000 rpm, 10 minutes), and plasma was stored at −20°C. Serum levels of coagulation markers—protein C, protein S, antithrombin III, fibrinogen, and homocysteine—were measured using BT-Labs kits (Shanghai Crystal Day Biotech Co.).

Statistical analysis was performed using SPSS v22. Data distribution was assessed using the Kolmogorov-Smirnov test. Normally distributed data were presented as mean ± SD; qualitative data were tabulated. The Mann–Whitney U test was used to compare quantitative data between IBD and control groups, given non-normal distribution. Kruskal–Wallis test assessed differences among the three subgroups (Crohn, ulcerative colitis, controls), followed by pairwise Mann–Whitney U tests. Categorical variables were evaluated using Chi-square or Fisher’s exact tests. To determine predictors of disease risk, both univariate and multivariate logistic regression analyses were conducted, with odds ratios (OR) and 95% confidence intervals (CI) reported.

## Results and Discussion

A total of 88 participants were included in this case-control study: 59 patients with confirmed IBD (23 with Crohn disease and 36 with ulcerative colitis) and 29 healthy controls. The two groups were matched for age and gender, with no significant differences observed (P = 0.418 and P = 0.690, respectively) (Table 1). Subgroup comparison between controls, Crohn disease, and ulcerative colitis also showed demographic homogeneity (P > 0.05).

**Table 1:** Demographic characteristics of the participants in control and case groups

Variable	Control (n=29)	Crohn (n=23)	Ulcerative Colitis (n=36)	P-value
Age (years)	38 ± 11	39.5 ± 14.1	41 ± 14	0.662
Male Gender (%)	38%	39%	44.4%	0.851

**Table 2:** Comparison of the level of Blood Coagulation Factors in IBD and non-IBD groups

Marker	Control (n=29)	Crohn (n=23)	Ulcerative Colitis (n=36)	P-value
Protein C (µg/ml)	2.78 (1.17–4.34)	0.89 (0.43–2.88)	1.83 (0.55–3.93)	0.023
Protein S (mg/L)	15.8 (9.15–27.25)	7.4 (4.6–9.9)	11.2 (5.25–22.12)	0.001
Antithrombin III	274.4 (192–1581.5)	111.4 (84–131.7)	128.8 (106–306.4)	<0.001
Fibrinogen (mg/ml)	1.6 (1.15–3.5)	1.0 (0.7–1.2)	1.45 (0.9–2.52)	0.003
Homocysteine (nM/ml)	36.1 (8.05–88.5)	5.5 (1.2–6.2)	6.15 (5–20.4)	<0.001

Analysis of serum coagulation markers using the Mann–Whitney U test revealed significant differences between the overall IBD group and controls across all five parameters. IBD patients had notably lower levels of Protein C, Protein S, Antithrombin III, Fibrinogen, and Homocysteine (all P < 0.05) (Table 2). 

Further stratification by diagnosis showed distinct patterns: the Kruskal–Wallis test indicated significant differences among Crohn disease, ulcerative colitis, and control groups for all five markers (P < 0.05 each).

Pairwise comparisons revealed that Protein S, Antithrombin III, and Fibrinogen were significantly lower in Crohn patients than in those with ulcerative colitis (P = 0.016, 0.009, and 0.017, respectively). Additionally, Crohn disease patients showed significant reductions in all five markers compared to controls (P < 0.01 for all), while ulcerative colitis patients differed from controls only in Antithrombin III (P = 0.003) and Homocysteine levels (P = 0.001).

To assess predictive utility, univariate logistic regression indicated that Protein S (OR = 1.123, 95% CI: 1.007–1.252, P = 0.038) and Fibrinogen (OR = 2.935, 95% CI: 1.089–7.915, P = 0.033) may help distinguish Crohn disease from ulcerative colitis. However, these associations did not retain significance in multivariate models.

When comparing IBD patients to healthy individuals, univariate analysis identified Antithrombin III (OR = 1.0, 95% CI: 0.999–1.0, P = 0.03) and Homocysteine (OR = 0.973, 95% CI: 0.958–0.987, P < 0.001) as significant indicators. In multivariate analysis, only Homocysteine remained independently predictive (OR = 0.957, 95% CI: 0.93–0.986, P = 0.003). 

In this cross-sectional study, 88 participants—including 59 patients with IBD (39% Crohn disease, 61% ulcerative colitis) and 29 healthy controls—matched for age and gender were analyzed to investigate alterations in coagulation factor levels. While the Mann–Whitney U test revealed significant differences in all five studied markers between IBD and non-IBD patients, multivariate logistic regression identified only homocysteine (OR = 0.957, 95% CI: 0.93–0.986, P = 0.003) as an independent predictor of IBD.

Various studies have explored blood coagulation factors in IBD, often reporting inconsistent findings. These discrepancies suggest that coagulation status in IBD may be driven by a multifactorial interplay of inflammation, genetics, endothelial dysfunction, and nutritional status, contributing to the variability among patients.

The incidence of thrombotic complications in IBD—particularly among young adults—is rising (14), yet their exact pathogenesis remains unclear. Not all patients with abnormal coagulation profiles experience thrombosis, and conversely, not all IBD patients with thrombosis display classical coagulopathies (15). In a cohort study by Algahtani et al., 8% of IBD patients experienced thrombotic events within one year, with family history emerging as the only independent risk factor, elevating risk approximately ninefold (16).

However, up to 50% of thrombosis cases in IBD occur without identifiable risk factors (17). Supporting this ambiguity, Jackson et al. reported normal levels of proteins C, S, and antithrombin III across IBD patients (18). Similarly, in a cohort of 1253 IBD cases, no deficiencies in protein C or S were observed, with only one patient exhibiting antithrombin III deficiency (19). These findings highlight the absence of a unifying pathophysiological mechanism and explain the lack of consensus on prophylactic strategies for thrombosis in IBD (20). 

Against this backdrop, our study aimed to characterize serum levels of five coagulation markers—protein C, protein S, antithrombin III, fibrinogen, and homocysteine—and compare them between IBD patients and healthy individuals. Our results confirmed significantly altered levels among IBD patients, particularly among those with Crohn disease.

Findings by Mokhtari et al. parallel ours, showing fibrinogen levels in ulcerative colitis patients similar to controls but significantly higher than in Crohn disease patients (21). Alkim et al. also observed reduced protein S and antithrombin III in IBD; however, they did not detect significant inter-subtype differences (23), which contrast with our findings. Regarding protein C, Owczarek et al. reported higher levels in IBD patients, especially those with ulcerative colitis, and associated protein C activity with disease state. They proposed dysfunction in the protein C pathway across both active and inactive phases as a contributor to hypercoagulability (4). Consistent with our results, Souto et al. noted decreased antithrombin III in IBD patients, reinforcing the presence of a prothrombotic state (3). We found lower levels of protein S and fibrinogen in Crohn disease relative to ulcerative colitis, which may reflect differences in nutrient absorption. Crohn disease involves the small intestine, where most amino acid absorption occurs, potentially impairing the synthesis of coagulation proteins. In contrast, ulcerative colitis has limited impact on nutrient absorption, which could explain the comparatively preserved protein levels.

Homocysteine findings in our study differed from previously published data. Oldenburg et al. documented higher homocysteine levels and hyperhomocysteinemia prevalence in IBD patients (23), whereas our results showed significantly lower levels in IBD subjects compared to controls. This contradiction may stem from differences in laboratory methods, study populations, and sample sizes. Other confounding factors, such as genetic variants (e.g., MTHFR mutations), deficiencies in folate and vitamins B6/B12, underlying diseases (e.g., renal failure, hypothyroidism, alcoholism), smoking, and medications (e.g., steroids, cyclosporine), could also affect homocysteine concentrations.

In summary, our study reinforces the idea that IBD is associated with diverse and significant changes in coagulation factor profiles. These alterations may vary by disease subtype and pathophysiology, especially in the context of nutritional impairment and inflammation. Homocysteine emerged as the strongest independent predictor among the measured factors, warranting further investigation into its clinical utility. While no definitive biomarker or preventive therapy for thrombosis in IBD has yet been established, profiling coagulation markers may offer valuable insight into individualized risk assessment and future therapeutic approaches.

Our findings indicate that inflammatory bowel disease (IBD) is associated with alterations in serum levels of key blood coagulation factors, contributing to a hypercoagulable state. Notably, antithrombin III levels were significantly lower in IBD patients compared to healthy individuals, supporting previous reports of increased thrombotic risk in this population. Surprisingly, homocysteine levels, which are typically elevated in coagulopathic conditions, were significantly reduced among IBD patients. This unexpected pattern may reflect differential physiological responses within coagulation and antioxidant systems under chronic intestinal inflammation.

These variations underscore the complexity of coagulation dynamics in IBD and suggest that a multifactorial mechanism governs individual susceptibility to thrombotic events. Given the clinical relevance of these findings, especially in stratifying thrombotic risk, further research is warranted to confirm and elucidate the observed trends.

Future studies should aim to establish population-specific reference ranges and diagnostic cutoff points for coagulation factors in both healthy individuals and IBD patients. Additionally, evaluating the feasibility and utility of routine coagulation screening in clinical practice may help improve prevention strategies, particularly among hospitalized or high-risk IBD patients.

Expanding the sample size, accounting for disease activity, nutritional status, and genetic factors, will be crucial in refining our understanding of coagulopathy in IBD and informing therapeutic decision-making. 

## Data Availability

All data generated during the study are included in this article. Further enquiries can be directed at the corresponding author.
